# Lower viral evolutionary pressure under stable versus fluctuating conditions in subzero Arctic brines

**DOI:** 10.1186/s40168-023-01619-6

**Published:** 2023-08-07

**Authors:** Zhi-Ping Zhong, Dean Vik, Josephine Z. Rapp, Olivier Zablocki, Heather Maughan, Ben Temperton, Jody W. Deming, Matthew B. Sullivan

**Affiliations:** 1 Byrd Polar and Climate Research Center, Ohio State University, Columbus, OH USA; 2 Department of Microbiology, Ohio State University, Columbus, OH USA; 3 Center of Microbiome Science, Ohio State University, Columbus, OH USA; 4 Department of Biology, Université Laval, Québec, QC Canada; 5 Center for Northern Studies (CEN), Université Laval, Québec, QC Canada; 6 Ronin Institute, Montclair, NJ USA; 7 School of Biosciences, University of Exeter, Exeter, Devon UK; 8 School of Oceanography and Astrobiology Program, University of Washington, Seattle, WA USA; 9 Department of Civil, Environmental and Geodetic Engineering, Ohio State University, Columbus, OH USA

**Keywords:** Arctic, Viruses, Subzero and hypersaline brines, Cryopeg brine, Sea ice brine, Long- and short-read viromics, Metatranscriptomics, Population genetics, Evolution, Gene transfer

## Abstract

**Background:**

Climate change threatens Earth’s ice-based ecosystems which currently offer archives and eco-evolutionary experiments in the extreme. Arctic cryopeg brine (marine-derived, within permafrost) and sea ice brine, similar in subzero temperature and high salinity but different in temporal stability, are inhabited by microbes adapted to these extreme conditions. However, little is known about their viruses (community composition, diversity, interaction with hosts, or evolution) or how they might respond to geologically stable cryopeg versus fluctuating sea ice conditions.

**Results:**

We used long- and short-read viromics and metatranscriptomics to study viruses in Arctic cryopeg brine, sea ice brine, and underlying seawater, recovering 11,088 vOTUs (~species-level taxonomic unit), a 4.4-fold increase of known viruses in these brines. More specifically, the long-read-powered viromes doubled the number of longer (≥25 kb) vOTUs generated and recovered more hypervariable regions by >5-fold compared to short-read viromes. Distribution assessment, by comparing to known viruses in public databases, supported that cryopeg brine viruses were of marine origin yet distinct from either sea ice brine or seawater viruses, while 94% of sea ice brine viruses were also present in seawater. A virus-encoded, ecologically important exopolysaccharide biosynthesis gene was identified, and many viruses (~half of metatranscriptome-inferred “active” vOTUs) were predicted as actively infecting the dominant microbial genera *Marinobacter* and *Polaribacter* in cryopeg and sea ice brines, respectively. Evolutionarily, microdiversity (intra-species genetic variations) analyses suggested that viruses within the stable cryopeg brine were under significantly lower evolutionary pressures than those in the fluctuating sea ice environment, while many sea ice brine virus-tail genes were under positive selection, indicating virus-host co-evolutionary arms races.

**Conclusions:**

Our results confirmed the benefits of long-read-powered viromics in understanding the environmental virosphere through significantly improved genomic recovery, expanding viral discovery and the potential for biological inference. Evidence of viruses actively infecting the dominant microbes in subzero brines and modulating host metabolism underscored the potential impact of viruses on these remote and underexplored extreme ecosystems. Microdiversity results shed light on different strategies viruses use to evolve and adapt when extreme conditions are stable versus fluctuating. Together, these findings verify the value of long-read-powered viromics and provide foundational data on viral evolution and virus-microbe interactions in Earth’s destabilized and rapidly disappearing cryosphere.

Video Abstract

**Supplementary Information:**

The online version contains supplementary material available at 10.1186/s40168-023-01619-6.

## Background

Earth’s cryosphere represents regions with frozen water, including ice-covered oceans and permafrost (frozen soils), a significant portion (~13%) of Earth’s surface [1]. These regions are inhabited by microbes of all three domains of life and dominated by psychrophiles well-adapted to these icy habitats where they provide key ecological functions [2]. Cryosphere microbes are of much interest for their potential in biotechnological applications [3], as analogs of extraterrestrial life [4], and in modeling ecological responses and the consequences of global warming [5]. With the cryosphere shrinking at unprecedented speed in response to climate warming [5] and the subsequent ecosystem losses, our opportunities to understand organismal biology, ecology, and evolution under the uniquely extreme conditions of the cryosphere are jeopardized.

Among the understudied cryosphere habitats are subzero hypersaline brines (kept in liquid phase below the freezing point of water by high salinity), which provide natural ecosystems to investigate the adaptive mechanisms of microbes and the evolutionary pressures they experience under very low temperatures and high salinities. Because evolutionary pressures in milder settings appear to differ depending on whether environmental conditions are constant versus fluctuating [6], we were motivated to conduct comparative studies of two different types of subzero brines: thermally stable cryopeg brine (CB; ancient brine found within permafrost) and temporally changing sea ice brine (SB; inherent to the sea ice of polar seas and oceans). The CBs we studied are believed to have originated from seawater-saturated marine sediments that were exposed to a freezing climate during past regression of the Arctic Ocean and then trapped beneath impermeable layers of permafrost, isolating the brines from the atmosphere and meteoric water for many thousands of years [7, 8]. Thus, CB represents an ancient marine habitat of relatively stable temperature (−8 to −6°C) and corresponding salinity (112–140 ppt salt for those we studied) over the long term [9–11]. In contrast, SB is a fluctuating habitat with temperatures and salinities that change seasonally and even daily [12], from extremes of −30°C and 240 ppt salt in winters to approximately 0°C and <0.5 ppt salt in summers [2, 13]. The brines within first-year sea ice are also ephemeral environments, forming from seawater during the fall freeze-up period and further concentrating seawater constituents (including microbes) through the winter and spring, then draining and diluting back into surface seawater as the ice melts in summer [14]. Therefore, both CB and SB are characterized by extreme conditions of low temperature and high salinity, while they differ substantially in formation process, age, and stability.

Despite the extreme conditions, both CB [7, 15, 16] and SB [2, 17] are inhabited by diverse and active microbial communities [10], which can potentially influence cryosphere responses to climate changes [18]. Previous work suggests that Gammaproteobacteria and Bacteroidia (more specifically, the genera *Marinobacter* and *Gillisia*) are dominant members of Alaskan CB, whereas the better-studied SB habitat has a high abundance of Flavobacteriia, Gammaproteobacteria, and Alphaproteobacteria (e.g., the genera *Polaribacter*, *Glaciecola*, and *Octadecabacter*) and higher microbial diversity than CB [2, 16, 19–22]. Metagenomic and metatranscriptomic analyses showed that the communities in both brine systems have evolved distinct adaptations to survive and even thrive under the prevailing environmental pressures in subzero and hypersaline brines [9–11].

Compared to microbes, viruses are largely underexplored components of these brine communities, although they presumably are able to impact microbes in subzero brines, as they impact microbes in marine planktonic habitats [23–26] via host cell lysis, gene transfer between hosts, and host metabolism reprogramming. To date, SB virus studies that involved quantification or cultivation have shown high viral concentrations and potentially high virus-host contact rates [27–31], while the only existing viral metagenomic survey hinted at their potential impact on microbial metabolism [32]. For CB, two studies [32, 33], each based on a single brine sample, showed that the brine was dominated by novel viruses, with viral communities of relatively low species richness compared to SB and that some of these viruses encoded an auxiliary metabolic gene (AMG), fatty acid desaturase gene, that might impart flexibility to the host cell membrane as an adaptation to cold and salty brines [32].

In spite of such progress, knowledge of virus-microbe interactions (e.g., via infection, gene transfer, and metabolism modulation) in CB and SB is very limited, with virtually nothing known about viral activity in CB or viral microdiversity (i.e., intra-species genetic variation) in either SB or CB. Evidence from a temperate hypersaline lake suggests that high ionic concentrations may increase microdiversity [34]. In our subzero brines, acquiring such information in a comparative sense can provide insights into how viral speciation, niche definition, and gene selection pressures [35] may differ between stably extreme versus fluctuating conditions. Here, we leveraged both Illumina short-read and Nanopore long-read sequences to generate viromes from extracellular viruses in Arctic cryosphere samples of CB, SB, and the underlying seawater (SW). With these viromes and two published metatranscriptomes (one each for CB and SB) from the same project [10], we explored viral community composition, environmental distribution, functional gene repertoire and potential gene transfer, transcriptomic activity, and evolution within these extreme environments. Using the long-read-powered viromes, we also comparatively evaluated evolutionary pressures that viruses have been experiencing under relatively stable (CB) versus fluctuating (SB) environmental conditions in these cold and hypersaline brines.

## Results and discussion

### Establishing a high-quality virus dataset

Four viromes were constructed from four Arctic cryosphere samples: two CB samples (labeled CB17 and CB18) collected from the same borehole site in successive years (2017 and 2018), one sea ice brine sample (labeled SB), and one seawater sample (labeled SW) from the same region (Figure S1). Two of them (i.e., CB17 and SB) were sampled in 2017 for comparing the viral community compositions using short-read viromes [32]. Here, we enhanced virus recovery from these samples via (results below): increasing sequencing (2.1 times deeper), adding long reads, upgrading assemblies, and using new methods that can better capture viral signals from short contigs. Combining the 2017 samples (CB17 and SB) with two new samples (CB18 and SW) collected in 2018, and using both short- and long-read viromes (named “long-read-powered viromes”), we generated a total of ~7.5 × 10^10^ quality-controlled bases of sequence data (Table S1), on average 2.6 times deeper per metagenome than the previous report of brine viromes [32].

**Fig. 1:**
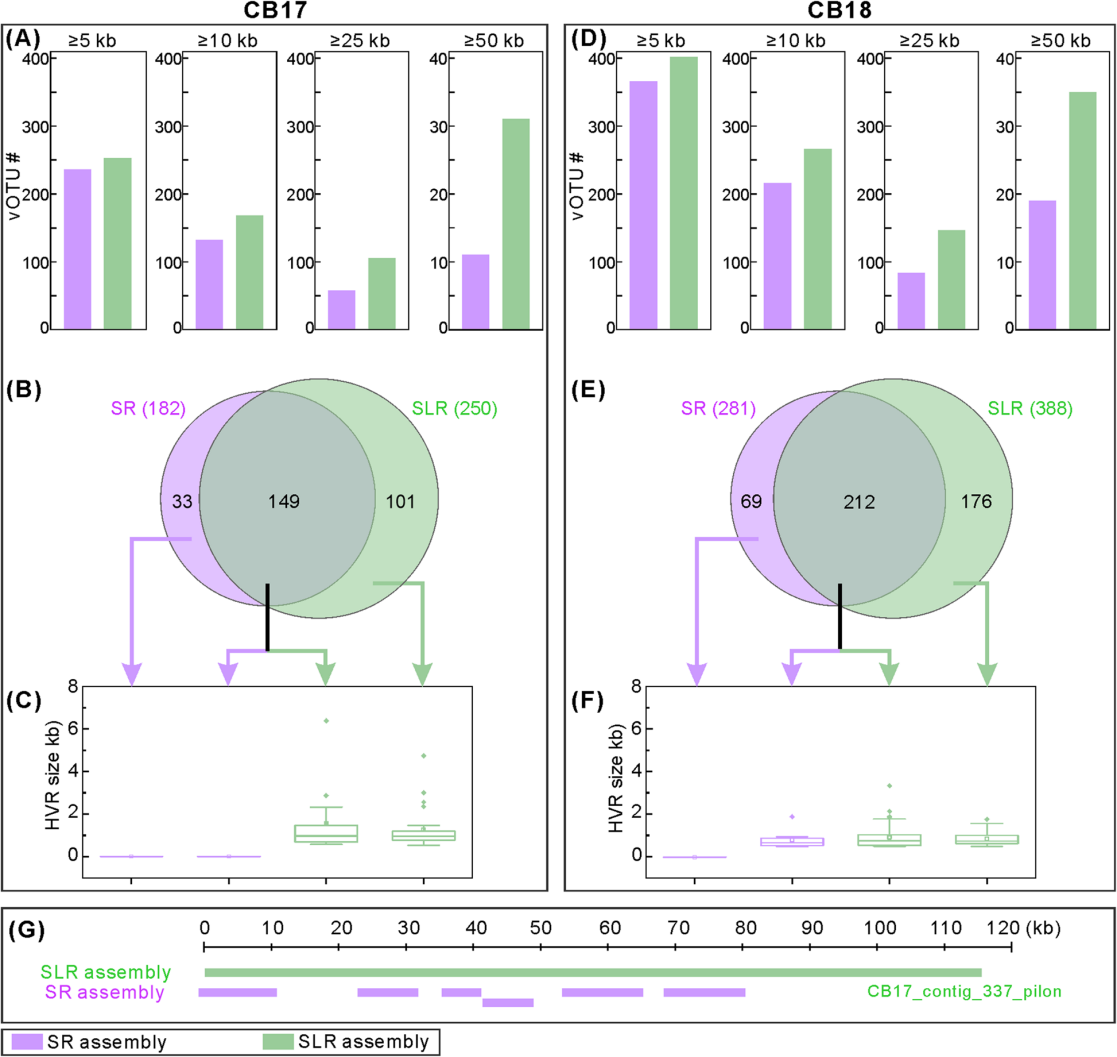
Recovery of vOTUs and their hypervariable regions (HVRs) from the paired short-read-only (SR) and short+long-read (SLR) assemblies from CB. Both assemblies were conducted with identical sequencing depth (i.e., identical number of nucleotides; see Table S2). **A–C** Comparison for sample CB17: **A** Number of vOTUs recovered from SR (lavender) and SLR (light green) assemblies by selecting vOTUs at lengths of ≥5, ≥10, ≥25, and ≥50 kb. **B** Number of unique and shared vOTUs between the two assembly types; viral contigs were clustered into a vOTU if sharing ≥95% nucleotide identity across ≥80% of their lengths. **C** HVR identification of all vOTUs including the unique and shared ones in both assemblies. More and longer HVRs were identified in vOTUs recovered from SLR assembly than SR assembly. **D–F** Comparison for sample CB18: **D**, **E**, and **F** display the same type of information as **A**, **B**, and **C**, respectively. **G** An example to show the genome matches of contig fragments recovered from SR assembly to the long contig (i.e., CB17_contig_337_pilon) recovered from SLR assembly. More examples of such comparisons are provided in Tables S3 and S4 for the vOTUs from the samples CB17 and CB18, respectively. SR assembly: only short reads from Illumina sequencing were used for assembly; SLR assembly: half depth of Illumina short reads and half depth of Nanopore long reads were used for hybrid and Pilon software-based assemblies (see Table S2 and “Methods”)

After generating the viromic dataset, we leveraged the low-input hybrid assembly approaches we developed previously [36, 37] to determine whether adding complementary long reads to short-read virome data would improve the recoveries of vOTUs (approximate to species-level viral operational taxonomic unit) and facilitate assembly of hypervariable regions (HVRs), which are informative for estimating microdiversity. Comparing “short-read-only (SR) assemblies” and “short+long-read (SLR) assemblies” (where both assemblies had the same sequencing depth) for the two CB samples indicated that an average of 9, 25, 77, and 133% more vOTUs of length ≥5, ≥10, ≥25, and ≥50 kb, respectively, were obtained in SLR than SR assemblies (Fig. 1A/D; Table S2), with N50 increasing from ~33 kb in SR to ~41 kb in SLR. In addition, SLR assemblies obtained ~3 times more unique vOTUs (Fig. 1B/E) and significantly improved (>5 times) the more challenging assemblies of HVRs (Fig. 1C/F). Genomic comparisons further revealed that 25% of contigs derived from SR assemblies were nested within contigs derived from SLR assemblies (Fig. 1G; Tables S3 and S4). Together, these results indicated that long-read sequencing significantly improved the recoveries of vOTUs (particularly for long viral contigs) and HVRs within viral genomes, consistent with previous findings [36, 37]. Here, however, we evaluated these improvements while controlling for differences in sequencing depth among assemblies, which was not done in other reports [36, 37] but can significantly influence such comparisons. To maximize virus recovery, we followed the established pipeline [36, 37] to use all reads (i.e., without subsampling) for assembly and combined viral contigs generated from both SR and SLR for further analysis.

A total of 11,088 vOTUs (≥5 kb) were recovered, including 6142 vOTUs ≥10 kb (Table S1). Among them, 5680 vOTUs (≥5 kb) were from CB and SB, a 4.4-fold increase over the 1305 vOTUs reported previously for the same ecosystems [32]. In addition, the N50 and the percentage of complete genomes (PCG, assessed by checkV) for the 11,088 vOTUs were 1.4 times longer (N50: 21.9 kb versus average 15.5 kb) and 2.0 times higher (PCG: 3.0% versus average 1.5%) than for vOTUs from previously reported CB/SB [32] and the Global Ocean Viromes 2.0 (GOV2) dataset [38]. In all samples, 78.1% (range 58.8–97.6%) of the reads recruited to the 11,088 vOTUs (Table S1). This level of read recruitment is exceptional and may represent the best recovery of any viromes published to date; compared to the also deeply sequenced GOV2 dataset, this read recruitment was about 4-fold higher [38]. Beyond the markedly strong read recruitment to reference genomes, rarefaction analysis also suggested that viral sampling was close to saturation (Figure S2), with less than 6, 11, and 50 additional vOTUs identified if adding an additional 1 million reads to the CB, SB, and SW libraries, respectively (See “Methods”). Overall, we generated four high-quality viromes for these largely understudied Arctic cryosphere brines and established an important virus dataset for further ecological and evolutionary investigations.

### CB communities remain mostly stable through two successive years

**Fig. 2:**
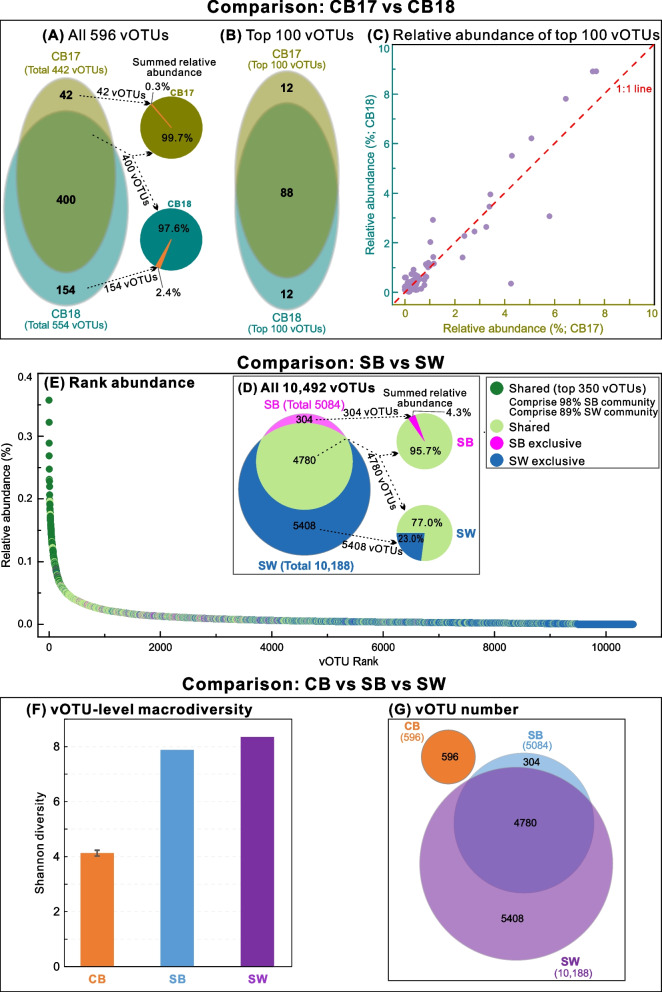
Viral communities and macrodiversity among CB, SB, and SW. **A–C** Comparison of microbial communities between CB17 and CB18, two cryopeg brine samples from successive years (2017 and 2018). **A** Venn and pie plots illustrate all the shared and unique vOTUs and their accounted relative abundances in the two samples. **B** Venn plot illustrates the shared and unique vOTUs based on the top 100 abundant vOTUs in the two samples. **C** Relative abundances of the top 100 abundant vOTUs in the two samples. **D–E** Comparison of viral communities in sea ice brine (SB) and seawater (SW). **D** Venn and pie plots illustrate all the shared and unique vOTUs and their accounted relative abundances in the two samples SB and SW. **E** Rank abundance curve shows the relative abundances of SB and SW vOTUs. The vOTUs were ordered according to their relative abundances in SW, with the addition of the exclusive vOTUs in SB based on their relative abundances. The shared and unique vOTUs are indicated by colors. **F** Viral macrodiversity (by vOTU-level Shannon diversity; the error bar indicates a standard error of the mean for the Shannon diversity of the two CB samples, i.e., CB17 and CB18) and **G** the number of shared and unique vOTUs among CB, SB, and SW ecosystems

We first compared the viruses between the two CB samples (CB17 and CB18) that were collected from the same permafrost borehole in successive years 2017 and 2018. The 2 years overlapped in the majority of CB vOTUs (400 of 596, comprising >97.6% of each community), despite a slight increase of the overall viral concentration in 2018 and the differences in rare vOTUs that in total comprised only 0.3–2.4% of each community (Fig. 2A–C; Figure S3 & Table S1). These minor differences may be attributable to the variations in host relative abundances, or the possible effects of chemical differences (e.g., extracellular polysaccharide content) and the in situ spatial heterogeneity of brines between years [16].

### SB and SW communities mostly overlap, but are distinct from CB communities

Comparing SB and SW communities found that the most abundant vOTUs were present in both samples (Fig. 2D/E). However, SW had 2.0 times higher richness (10,188 vs 5084 vOTUs) than SB and the unique vOTUs were mainly rare viruses that totally comprised only 23.0% and 4.3% of the SW and SB communities, respectively. This result was expected, as seawater is the primary source of viruses (and their hosts) to brines in sea ice during the freeze-up, yet further selection by the harsh conditions in the brine may have reduced richness [17]. Because the viral communities were highly similar and closely linked environmentally, the SB and SW viromes were merged into one “SB/SW virus dataset,” just as the two CB viromes (CB17 and CB18) were combined into one “CB virus dataset” for subsequent comparative analyses (unless otherwise stated, as for viral concentration and diversity comparisons).

We then assessed the viral concentration, diversity, and community composition in CB to those in SB/SW. As published previously [16, 32], viral concentration in CB was about 3 orders of magnitude higher than in SB and SW (i.e., 10^8^ versus 10^5^ mL^–1^; Table S1), while viral diversity was substantially lower than in either SB or SW (Shannon: 4.1 ± 0.1 for CB versus 8.0 ± 0.2 for SB/SW; Fig. 2F). This pattern was consistent with the findings for microbes in the same samples and might be partly due to the higher organic matter (~100 times higher; more organic matter may support more organisms) and salinity (~2 times higher; higher salinity selects for a smaller number of adapted members, thus lower diversity) in CB than SB/SW [16]. Read recruitment-based analysis found that no vOTU was shared between CB and SB/SW, indicating that CB contained unique viral species (Fig. 2G). Dating of the permafrost matrix in which these CB are embedded suggested that the CB and their viruses (and hosts) have been isolated from the atmosphere and meteoric water for at least 40,000 years [8], which would have provided a long time period for viral communities to diverge. This description of the CB viral community expands the known diversity of viruses in the cryosphere by adding entirely unique members not found in SB or SW, and further strengthens our previous efforts [32] by increasing the number of discovered brine vOTUs fourfold.

### Higher novelty in CB viruses and potential ancient marine origin

**Fig. 3:**
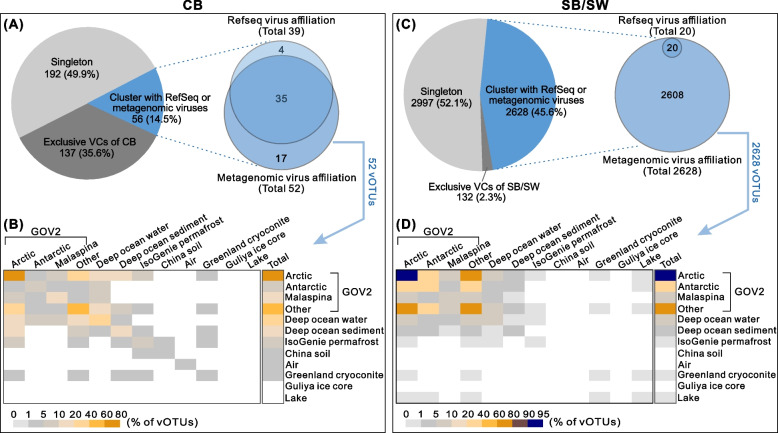
Genus-level profiles and global distribution. **A**, **C** Genus-level profiles of CB and SB/SW vOTUs, respectively. The genus-level profiles were constructed by the genome-content-based network analysis of vOTUs from this study, viral genomes from the NCBI RefSeq database, and vOTUs from 250 environmental metagenomes in the following ecosystems: global oceans (GOV2 dataset), deep ocean water, deep ocean sediment, surface layers of permafrost (IsoGenie), soil, air, glacier cryoconite, glacier ice core, and lake water (see “Methods”). SB and SW viruses were combined for the analysis (as SB/SW). **B**, **D** Environmental associations of CB and SB/SW vOTUs, respectively. For the CB (**B**) and SB/SW (**D**) vOTUs that clustered (i.e., shared VCs/genera) with viruses from environmental metagenomes, the distribution of environmental habitats was evaluated and illustrated by a heatmap. The gradient colors in the heatmap represent the percentage of vOTUs (weighted by the associated vOTUs) that were associated with the different ecosystems. Each cell in the last column (i.e., labeled as “Total”) indicates the total percentage of vOTUs detected from each of the tested environments on the right-side legend, while each cell in the other columns (i.e., the 12 columns in the left rectangle) indicates the percentage of vOTUs that were detected from both of the two environments suggested at the top- and right-side legend of the heatmap. For example, the cells associated with both Arctic and Deep ocean water showed that 10–20% (**B**) and 5–10% (**D**) of the 52 CB and 2628 SB/SW vOTUs, respectively, were detected from both of the Arctic and Deep ocean water environments. Most of the CB and SB/SW viruses were associated with seawater viruses from the Arctic

We next evaluated the genus-level novelty and environmental distribution of all 6142 longer vOTUs (≥10 kb) by genome-based network analyses to compare them to published viruses in the RefSeq database and 250 metagenomes from many habitats: global oceans (GOV2), deep ocean water and sediment, permafrost, soil, air, glacier cryoconite and ice core, and lake water (see “Methods”). First, only one viral cluster (VC; approximate genus-level taxonomy) was shared between the CB and SB/SW samples (Table S5), implying that each community type had distinct viral genera. Second, 14.5 and 45.6% of the CB and SB/SW vOTUs, respectively, formed VCs with viruses from databases (Fig. 3A/C; Table S5 and Figure S4), indicating a much higher (85.5% versus 54.4%) genus-level novelty of viruses in CB than SB/SW. Third, for those vOTUs associated with database viruses, most of them (73.1 and 92.4% for CB and SB/SW, respectively) formed VCs with the GOV2 Arctic seawater viruses (Fig. 3; Table S5). The implication at the genus level that some CB viruses originated from seawater (prior to becoming isolated within permafrost) supports the marine origin of CB in the Utqiaġvik region, previously based on salt composition, stable isotopes, the presence of *Marinobacter*-like viral genes, and an abundance of *Marinobacter* species in the brines [8, 16, 33].

Given that genus-level analyses showed that most (84.6 and 99.9% for CB and SB/SW, respectively) of the database-affiliated vOTUs were associated with the GOV2 sample-derived viruses (Fig. 3; Table S5), we further leveraged these GOV2 metagenomes to evaluate the global distribution of CB and SB/SW viral species by recruiting metagenomic reads to the vOTUs (≥5 kb) from both this study and GOV2 datasets (see “Methods”). Similar to the findings from genus-level analyses, only a single vOTU was shared between CB and GOV2 samples, while SB/SW samples comprised viruses that were mostly related to Arctic viruses, as indicated by nonmetric multidimensional scaling (NMDS) analysis (Figure S5).

### Viruses predicted to actively infect the dominant brine microbes

**Fig. 4:**
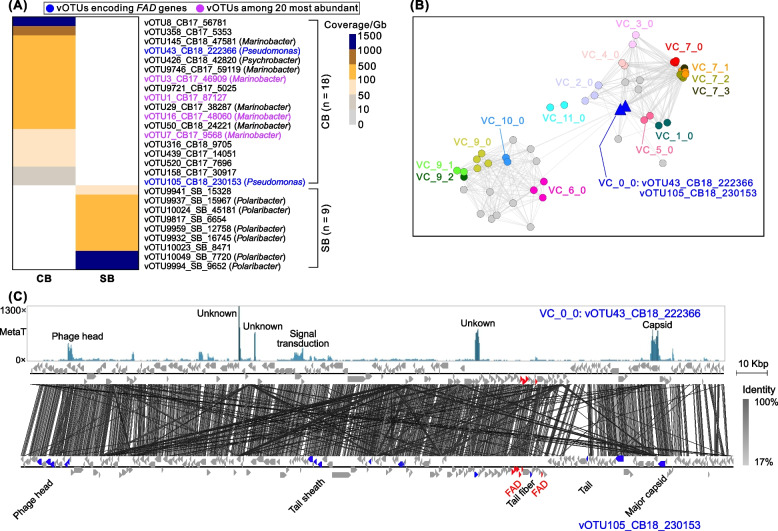
Potential activity of brine vOTUs by metatranscriptomic read recruitment. **A** Coverage of active vOTUs based on metatranscriptomic read mapping to virome-generated vOTUs from CB and SB samples. The metatranscriptomic reads were mapped (90% read identity + 90% read coverage + 50% contig coverage) to brine vOTUs to identify potentially active viruses. The potential active vOTUs that contained the AMG *FAD* genes and that were among the top 20 abundant viruses (compared to the whole community that comprised both active and inactive viruses) were marked in blue and purple, respectively. The two vOTUs containing the AMG *FAD* genes did not belong to the top 20 abundant viruses. **B** Network analysis shows the genus-level taxonomy of the two vOTUs containing the AMG *FAD* genes. These two vOTUs belonged to the same VC (i.e., approximate to a viral genus) and are indicated in blue triangles. **C** The genome composition and the coverages of mapped metatranscriptomic reads for the two vOTUs encoding the AMG *FAD* genes. The vOTU vOTU43_CB18_222366 was recovered with a complete (circular) genome, while vOTU105_CB18_230153 had 85% completeness based on checkV assessment. The AMG *FAD* genes are indicated in red, while the phage structural genes, including head, tail, and capsid genes, are indicated in blue. All other genes are colored in gray. Genes with high activity (i.e., interpreted by high reads coverage; *top panel*) included phage structural genes (i.e., head and capsid), signal transduction gene, and some unknown genes. The *FAD* genes were active at a low level

We next investigated whether there were gene transcriptions from brine viruses while in their hosts, either as infecting lytic viruses or as prophages. To address this question, published metatranscriptomic reads from the same project [10] were recruited to the 596 CB and 5084 SB vOTUs. The results revealed that 18 CB and 9 SB vOTUs were potentially active in the brines, with 7 of the 18 active CB vOTUs and 6 of the 9 active SB vOTUs predicted to infect *Marinobacter* and *Polaribacter*, respectively (Fig. 4A). These taxa were the dominant genera in their respective brine types [10, 16], suggesting that viruses have been actively impacting the brine ecosystems through infection of the dominant microbial lineages. As well, two CB vOTUs that encoded fatty acid desaturase (*FAD*) genes, thought to improve host survival via membrane adaptation to the brine environment [32], were also active. Through the additional sequencing here, the genomic context of the two vOTUs was extended to 1.7 and 9.2 times longer (i.e., 222 and 230 kb versus 127 and 25 kb, respectively). These two *FAD*-encoding viruses were predicted to infect *Pseudomonas*, belonged to the same viral genus (likely a novel one; Fig. 4A/B; Table S6), and had highly similar genomes (Fig. 4C).

We then explored which genes were being transcribed by these two *Pseudomonas* phages, according to the coverage of recruited reads from metatranscriptomes, and found that a capsid gene, a head gene, a gene relevant to signal transduction, and several genes of unknown function were among the genes that were most actively transcribed (i.e., with highest metatranscriptomic read-based coverage). The *FAD* genes had some representative reads but with lower coverage (Fig. 4C), suggesting that the *FAD* genes might be transcribed at low levels or only under certain conditions or certain infection stages (e.g., not during virion assembly, the putative occurrence of which in these samples was suggested by the high transcription of capsid and head genes in the two *Pseudomonas* phages; Fig. 4C). Overall, though only a small number of active viruses were identified, these results from metatranscriptomic analyses provided the first window into the activity of CB viruses—at both the genome and gene level—and reinforce our understanding that viruses likely impact brine ecosystems through active infection of abundant microbes and encoding host-derived metabolic genes.

### EpsG: a novel AMG potentially influencing host EPS synthesis in CB

**Fig. 5:**
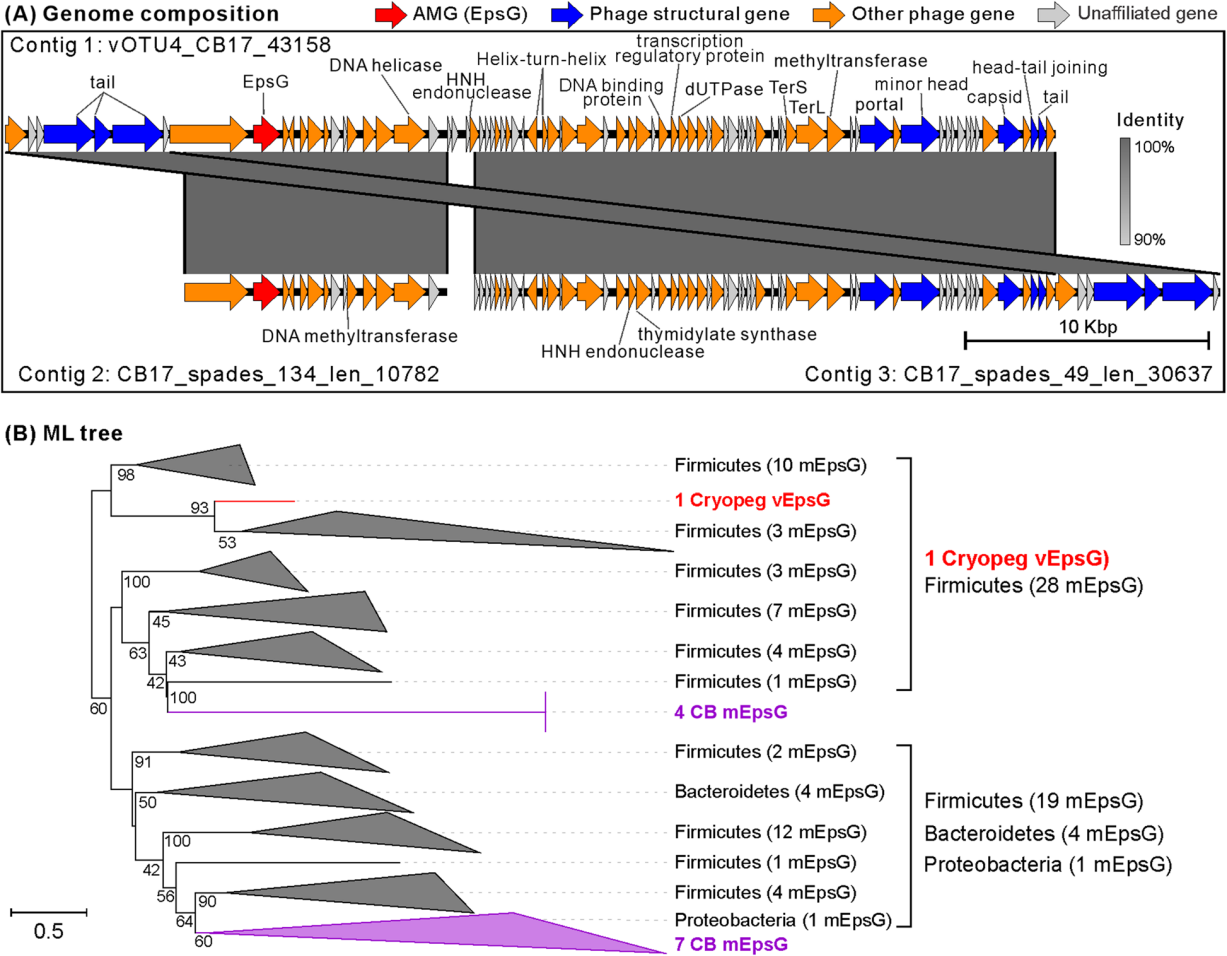
Characterization of the virus-encoded auxiliary metabolic gene (AMG) *epsG*. **A** Genome map of the CB virus vOTU4_CB17_43158 encoding *epsG*. This vOTU had a circular genome due to the overlapped regions between two ends. The full genome was recovered by long-read assembly, while the two contig fragments, which had 100% DNA identity to the full genome, were recovered by SR assembly. CheckV was used to assess host-virus boundaries and remove potential host fractions on the viral contig (no host contamination was found; no cellular gene, except the AMG, was identified; see “Methods”). Genes were marked by four colors to illustrate AMGs (red), phage structural genes (blue), other phage genes (orange), and unaffiliated genes (gray). AMGs were detected by DRAM-v and following manual inspection; phage genes were classified by comparing their predicted protein sequences to those of a large database of 15,958 profile hidden Markov models by CheckV and of viral genes in the extended RefSeqABVir database by VirSorter v1 in virome decontamination mode. Genes were also annotated by comparing them to UniRef, PFAM, and KEGG databases. Genes were marked as “phage genes” if they were matched to the genes of viruses in the RefSeqABVir database or CheckV databases. Genes were considered “unaffiliated genes” if they had no hit to a sequence in any tested databases. **B** Phylogenetic tree of the viral and microbial *epsGs*. The tree was inferred using maximum likelihood (ML) method with EpsG protein sequences (see “Methods”). Bootstrap values (expressed as percentages of 1000 replications) of ≥40 are shown at the branch points. The scale bar indicates a distance of 0.5. The vEpsG sequence is indicated in red. The mEpsG sequences from the CB microbial metagenomes [10] and the NCBI nr database are indicated in purple and black, respectively. The full phylogenetic tree without collapse is provided in Figure S6

In addition to the *FAD* genes [32], we assessed whether the brine viruses encoded any AMG that could influence the production or metabolism of extracellular polysaccharides (EPS), which can serve as cryo- and osmo-protectant for microbes [39–43] and were >1000-fold more concentrated in CB than in SB and SW [16]. From gene annotations of all the recovered vOTUs (Table S7), we identified an *epsG* gene that was flanked by viral genes on a complete viral genome recovered from CB (Fig. 5A; Table S8). The genome was assembled from the long-read dataset, while the SR assembly yielded two fragments that were identical to the full genome (Fig. 5A). The *epsG* gene represents a novel AMG, as it has not been found previously to be encoded by viruses. In bacteria, the *epsG* gene is a member of the *eps* operon (gene cluster), which produces proteins that function in polymerization [44] for EPS synthesis and biofilm formation [45, 46]. Mutations in *epsG* can control EPS production and, in *Pseudoalteromonas atlantica*, biofilm formation [47, 48]. In subzero brines, microbes have been reported to produce EPS as both cryo- and osmo-protectants [39, 42]. We speculate that viruses hijacked this *epsG* to influence EPS synthesis by their hosts and thus enhance host survival in these harsh settings—a useful strategy for a prophage lifestyle.

We then explored the evolution and functionality of *epsG*. Phylogenetic analysis was conducted to evaluate the relationship of this virus-encoded *epsG* (*vEpsG*) to 61 microbial *epsG* (*mEpsG*) sequences that were recovered from the concurrently sampled CB microbial metagenomes [10] (*n* = 11) and from the NCBI nr database (*n* = 50). The CB *vEpsG* clustered with *mEpsGs* from the Firmicutes (Fig. 5B; Figure S6), indicating that the *espG* might have been transferred from a microbe belonging to the Firmicutes. An evaluation of the amino acid and protein sequences suggests that the vEpsG is functional. No conserved motif has been reported previously for EpsG, but we identified two motifs that were highly conserved in the tested vEpsGs and mEpsGs (Figure S7). Further, the number of nonsynonymous mutations relative to the number of synonymous mutations, a measure of selection pressure, was determined by recruiting short metagenomic reads from CB samples to this *vEpsG*. No single-nucleotide polymorphism (SNP) was discovered for the *vEpsG* gene, which thus was probably under purifying selection in CB, indicative of a functional gene. This interpretation was supported by analyzing the evolutionary dynamics of *epsG* homologs across lineages, which implied that the *epsG* was under purifying selection (average *dN/dS* = 0.053) and remained functional (Table S9). No activity was observed for this AMG by metatranscriptomic read recruitment, which might be due to transcription of *epsG* being too low for detection or only occurring under certain conditions. Though experimental evaluation is required to establish function, the genomic evidence that *vEpsG* is likely functional leads us to presume that it would alter host EPS synthesis in ways that improve cryo- and osmo-protection in the CB ecosystem.

### Lower viral evolutionary pressure in CB than SB/SW

**Fig. 6:**
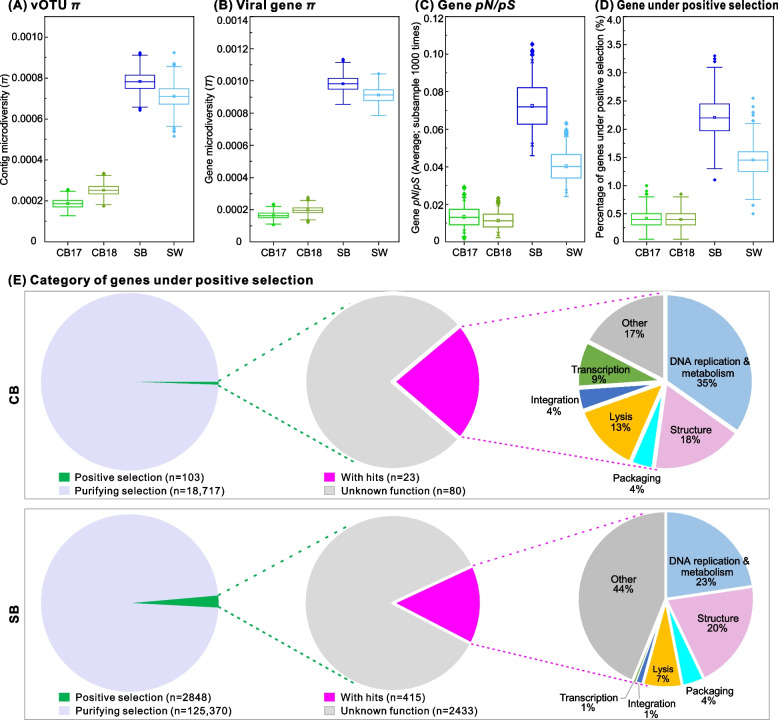
Microdiversity and positively selected genes. **A** Genome-level microdiversity indicated by *π* values. **B** Gene-level microdiversity indicated by *π* values. **C** Selection pressures of viral genes indicated by *pN*/*pS* values. **D** Percentage of genes under positive selection (i.e., *pN*/*pS* > 1). **E** Categories of viral genes under positive selection in cryopeg brine (CB) and sea ice brine (SB). *Left pie graph*, all genes are divided into two groups: genes under positive selection and purifying selection based on *pN*/*pS* values. *Middle pie graph*, genes under positive selection are divided into annotatable and un-annotatable groups by comparing to genes in databases. The number of genes in the above groups is indicated in parentheses after the group names. *Right pie graph*, the annotatable genes from the middle pie graph are grouped into different gene categories. The percentage of genes assigned to different categories is indicated after the category name. Sample labels: CB, cryopeg brine; SB, sea ice brine; SW: seawater

Intra-population variations (i.e., microdiversity) can improve ecological resilience and offer windows into population- and gene-level selective pressures [49–52]. With the relatively recent opportunity to calculate such variations in viromics [53], we next assessed whether viral microdiversity and the selection pressures acting on viral genes differed in the two subzero brine types, representing relatively stable (CB) and fluctuating (SB) conditions, with SW as a reference. This revealed that viral microdiversity (via nucleotide diversity *π* value and the density of SNPs at both genome and gene levels) and gene selection pressure (via *pN*/*pS*) in CB were significantly lower than in SB and SW (Fig. 6A–D; Figure S8). Higher microdiversity may be generated and maintained by species adaptation and expansion into harsh or disturbance-prone environments, in which the viruses may experience strong selection pressure; such processes may drive viral speciation and provide advantages for viral adaptation to environmental extremes or perturbations [35, 54]. Indeed, higher microdiversity reflects the increase in microbial stress responses [55–57] and adaptations to environmental fluctuations [58]. In this study, SB was collected from first-year sea ice that forms and melts annually and, during its lifetime, provides interior liquid habitats that fluctuate considerably with seasonal and diurnal changes in temperature and salinity, as well as in the composition of microbial communities [12, 16, 17]. In such systems, a higher level of microdiversity for a viral population could be advantageous because it may allow populations to survive when environmental conditions and hosts change [59]. In contrast, the studied CB has been separated from the surface environment and remained under relatively stable temperature and salinity conditions for millennia [8]. In such geophysically stable ecosystems, viruses might have become dominated by those best adapted to these unique brine habitats and be under a relatively relaxed environmental selection, as was also suggested for their microbial hosts [9, 11, 16].

Looking specifically at gene selection pressures (via *pN*/*pS*) in the two brine types, we found that 103 (0.5% of total 18,820) CB and 2848 (2.2% of total 128,218) SB viral genes were under positive selection (Fig. 6E). Functional gene annotation could be assigned to 23 of the 103 CB genes, and to 415 of the 2848 SB genes (Fig. 6E; Table S10). About half of these annotated and positively selected genes were related to DNA replication, metabolism, and virion structure (Fig. 6E)—genes often under strong selection pressure during adaptation to new microbial hosts [60, 61]. Because 80–85% of the positively selected genes had unknown functions, exploring the functionality of these positively selected genes at a large-scale level is challenging.

Though many functions remain to be more deeply explored, especially as annotations are improved, here we focused on phage tail fiber genes as a proxy for assessing absorption and infection of host cells [62], and thereby the phage-host co-evolutionary arms race [63–66]. We hypothesized that mutations in some tail fiber genes may be under positive selection during arms race evolution and more frequent in the fluctuating SB environment than in the relatively stable CB. A total of 707 phage tail fiber genes were identified from SB, including 23 genes (3.3% of 707) that were putatively under positive selection (*pN*/*pS* range 1.09–8.97; Table S10). In contrast, none of the 51 tail fiber genes identified from CB viruses had a *pN*/*pS* ratio greater than 1, implying that all were under purifying selection (Table S10). Finding a lower positive selection pressure for mutations in tail fiber genes in cryopeg compared to those in SB is consistent with our overall result of lower gene-level selection pressure in CB than SB (Fig. 6C/D) and further supports our proposition that viral evolutionary pressure was lower in the relatively stable CB habitat than in the fluctuating, transient habitats within sea ice.

## Conclusions

The Anthropocene marks an unprecedented era of human-impacted climate change that is altering ecosystems across the globe, and most dramatically in the cryosphere. These most impacted regions also tend to be the least studied, as they are remote and inhospitable to humans. The results presented here build upon prior work to provide a baseline understanding of how viruses in the cryosphere can impact ecosystem processes and how they, in turn, are impacted by the relative stability of the environmental conditions. Using recent sequencing and analytical advances, we explored CB and SB viruses at levels both between and within populations, which helps to understand their population-level selection and genes driving niche differentiation. These observations varied by the environment, despite both CB and SB being extreme in temperature and salinity, as the resultant viral communities were taxonomically distinct at both genus and species levels and under different selective pressures for presumably a large fraction of the last 40,000 years. As cryopeg environments begin to destabilize under climate change, both their viral and bacterial communities will be subjected to a much greater degree of environmental fluctuation than experienced since the late Pleistocene. The insights gained here thus reinforce knowledge of selective pressures under stable versus fluctuating conditions in moderate environments, expand the field to include extreme and climate-threatened habitats of the cryosphere, and suggest that higher selective pressures lie in the future for both CB viruses and their hosts.

## Methods

### Site characterization and field sampling

The cryopeg brine (CB17 and CB18; brine within permafrost), sea ice brine (SB; brine within sea ice), and seawater (SW) samples were collected in May 2017 and May 2018 near Utqiaġvik, Alaska (Figure S1). The detailed sampling procedures for CB and SB were described previously [8, 16, 32]. Briefly, CB was sampled by drilling a borehole using a cleaned and ethanol-rinsed ice auger to ~1.5 m below the floor of the Barrow Permafrost Tunnel in 2017 (71.2944° N, 156.7153° W; as illustrated previously [32] in their Fig. 1C). The available brine in the borehole was pumped into a sterile polypropylene bottle using a specialized apparatus consisting of hand pump, sterile vacuum flask, and sterile tubing in 2017, leading to a collection of about 500-mL liquid brine (i.e., sample CB17). New brine drained into the borehole after 1 year, leading us to collect another ~500-mL sample of brine in 2018 (i.e., sample CB18) using the same methods. The sample SB was collected from landfast first-year sea ice near the Barrow Sea Ice Mass Balance site operated by the University of Alaska Fairbanks [67] in May 2017 (location: 71.3730° N, 156.5047° W; Figure S1). The sampling site was covered by 16–19 cm snow which was removed prior to drilling the sackhole to 75 cm in depth (partial core hole; the sea ice was total about 110 cm in thickness) for brine collection. After brine drained into the sackhole for about 4 h, approximately 9 L of brine was collected into an acid-rinsed (1M HCl) 10-L cubitainer by manual pump. The sample SW (about 20 L) was collected in May 2018 (location 71.4730° N, 156.7294° W; Figure S1), using an acid-rinsed (1M HCl) 20-L cubitainer by manual pump through a sea ice hole from which the surface snow (6–10 cm deep) and a full ice core (about 110 cm in length) had been removed. All samples were transported in an insulated cooler to a −6°C cold room at the Barrow Arctic Research Center (BARC) and processed in a 4°C cold room within 6 h.

### Sample processing, viral counting, and metagenomic DNA extraction

Each sample was passed through a polycarbonate 0.22-μm-pore-size filter (Cat No. GTTP02500, Isopore) to remove cells and particles >0.22 μm. Cells captured on the filters were subjected to DNA extraction and microbial metagenomic sequencing for other studies in the same project [10, 16]. This study focused on extracellular viruses in the filtrate. The virus-like particles were counted using the wet-mount method [68]. For the samples SB and SW, viruses were concentrated using an iron chloride flocculation method [69] and stored at 4°C at the BARC. All samples were shipped at 4°C from Utqiaġvik to Ohio State University in Columbus, where samples were stored at 4°C until DNA isolation.

Viral genomic DNA was isolated according to the methods previously described [70]. Briefly, the viral concentrates of SB and SW were resuspended with ascorbic-EDTA buffer (0.1 M EDTA, 0.2 M MgCl_2_, 0.2 M ascorbic acid, pH 6.0). All samples were then treated with DNase I (100 U/mL) to eliminate free DNA and 100 mM EDTA+100 mM EGTA to halt DNase activity. Samples were then concentrated by using a 100-kDa Amicon centrifugal device (Cat. no. UFC910096, Millipore) where each sample was resuspended 3 times in ~800 μL (total volume) of ascorbate-EDTA buffer. DNA was extracted using Wizard^®^ PCR Preps DNA Purification Resin and Minicolumns (Cat. No. A7181 and A7211, respectively; Promega, USA) [70].

### Short- and long-read virome sequencing

Following DNA extraction, short-read virome sequencing was performed at the Joint Genome Institute (JGI), Department of Energy, USA. The DNA libraries were prepared using the Nextera^®^ XT Library Prep Kit (Cat No. 15032354, Illumina) with 12 cycles of PCR amplification to increase template concentrations in each library, according to the manufacturer’s instructions. All libraries were sequenced with the Illumina NovaSeq platform (2 × 150 bp). The Nanopore long-read virome library preparation and sequencing were performed according to a low-input DNA protocol [36, 37] that was designed for viral community sequencing. Briefly, the DNA was first sheared to 10–15-kb fragments and PCR-amplified following the manufacturer’s library preparation protocol (ligation sequencing kit SQK-LSK109, Oxford Nanopore Technologies). The libraries were sequenced by a MinION device for 48 h using a R9.4 revD flow cell.

All cryopeg and seawater samples yielded short- and long-read sequences. Although we collected two SB samples (one in 2017 and another in 2018), we successfully obtained “short-read sequences” only for the sample collected in 2017. Thus, for SB analyses, this study only used a paired short-read virome for the sample recovered in 2017.

### Virome analysis and characterization of viral communities

All metagenomic analyses were performed with the Ohio Supercomputer Center [71], except for the short-read viromes that were trimmed and filtered for quality by JGI using the previously established standard pipeline (see Table S11) [72]. The long-read viromes were processed based on the methods described in the VirION2 pipeline [36]. Briefly, raw reads were converted into fastq format using Guppy v2.3.1 (provided by Oxford Nanopore). Reads were then filtered with NanoFilt 2.2.0 [73] for quality (only reads with a PHRED score >9) and size (<1-kb reads were discarded).

After quality filtering, the viromic sequence data was assembled using three strategies within the VirION2 pipeline [36]. First, the Flye assembler v2.5 [74] was used to assemble the long reads into contigs, which were then error-corrected by Pilon v1.23 [75]—“Pilon assembly”. Second, both short and long reads were used as input to generate hybrid assemblies using metaSPAdes v3.13.2 (using the --nanopore –meta settings) [76]—“Hybrid Spades assembly”. Third, short reads only were assembled to contigs using metaSPAdes v3.13.2 (-k 21,33,55 --meta) [76]—“Spades assembly”. The assembled contigs (length of ≥5 kb or circular contigs with length of 1.5–5 kb) were then used to predict viral contigs using three tools: VirSorter v1.1.0 [77], DeepVirFinder v1.0 [78, 79], and MARVEL v0.2 [80]. Contigs were classified as viruses if they met one of the following four criteria: (i) Categories 1, 2, 4, or 5 of VirSorter v1.1.0; (ii) DeepVirFinder score of ≥0.9 and *p* < 0.05; (iii) MARVEL probability score of ≥90%; or (iv) DeepVirFinder score of ≥0.7 and *p* < 0.05 and MARVEL probability score of ≥70%. Viruses identified by all methods and assemblies were combined for all analyses in this study except for constructing the rarefaction curves and comparing between paired short- and long-read assemblies described below.

Viral contigs were first checked for contaminants by comparing them to viral genomes considered as putative laboratory contaminants (e.g., phages cultivated in our laboratory, including *Synechococcus* phages, *Cellulophaga* phages, and *Pseudoalteromonas* phages) using Blastn. The remaining contigs were clustered into vOTUs if they shared ≥95% nucleotide identity across 80% of their lengths as described previously [81, 82]. The longest contig within each vOTU was selected as the seed sequence to represent that vOTU. A coverage table of each vOTU was generated using iVirus’ BowtieBatch and Read2RefMapper tools by mapping quality-controlled reads to vOTUs, and the resulting coverage depths were normalized by library size to “coverage per gigabase of virome” [83, 84]. Rarefaction curves of viromes were produced by estimating vOTU (length of ≥5 kb) numbers as a function of sequencing depth (i.e., read number), which was obtained by subsampling quality-controlled reads (Figure S2). The last four subsampled libraries in each sample were used for constructing the slopes of curves and further predicting the number of vOTUs that would be generated with additional sequencing.

To technically compare the short- and long-read assembly strategies (i.e., Pilon, Hybrid Spades, and Spades assemblies), we subsampled identical sequencing depths for each assembly using the two CB samples (CB17 and CB18; Table S2). For example, we subsampled 4.6 billion short-read and 4.6 billion long-read bases (total = 9.2 billion bases) for both Pilon and Hybrid Spades assemblies (short+long-read assemblies; SLR assemblies) and subsampled 9.2 billion short-read bases for the Spades assembly (short-read-only assembly; SR assembly) for CB17 (Table S2). The vOTUs were obtained according to the methods described in preceding paragraphs and compared among assemblies regarding the number of vOTUs that were selected by four different length thresholds of ≥5, ≥10, ≥25, and ≥50 kb (Table S2), as well as the shared and unique vOTUs observed by vOTU clustering described above. In addition, the hypervariable regions (HVRs) were detected from vOTUs for comparisons across assemblies according to the methods described previously [36, 37]. Specifically, short reads were mapped to vOTUs using Bowtie 2 v.2.3.3.1 [85] to generate a bam file, from which the per-base coverage was calculated using BEDTools “genomecov” v.2.25.0 [86]. Subsequently, the per-base coverage values were parsed to identify genomic islands according to the following criteria: (i) the fraction of the median coverage was ≤20%, (ii) the genome region was at ≥500 bp in size, and (iii) the viral contig had ≥5× coverage.

To explore the environmental distribution of brine viruses, we compared the vOTUs in this study to viruses in the National Center for Biotechnology Information (NCBI) RefSeq database (release v85) and to 250 published environmental metagenomes, through a genome-based network analysis to group the viruses into viral clusters (VCs; one VC approximates one viral genus) using vConTACT v2.0 [87]. The 250 metagenomes were from a wide range of environments covering global oceans (GOV 2.0) [38], deep ocean water and sediments [88], soils [89, 90], lakes [91, 92], deserts [93–96], air [97, 98], cryoconite [99], Greenland ice sheet [99], and glacier ice cores [100].

The putative virus–host linkages were predicted in silico using three methods based on: (i) nucleotide sequence composition, (ii) nucleotide sequence similarity, and (iii) CRISPR spacer matches, as described previously [25, 100, 101]. The metagenome-assembled genomes (MAGs) obtained from CB and SB [10] in the same project as this study were used as the database to link viruses to their hosts. Briefly, the vOTUs from this study were linked to their microbial hosts using the oligonucleotide frequency dissimilarity measure by VirHostMatcher, with a dissimilarity score of ≤0.1 and possibility of ≥80% as the threshold to pick the host [102]. In addition to sequence composition analysis using VirHostMatcher, the nucleotide sequence of each vOTU was compared (Blastn) to MAGs, and the viral sequences were considered for successful host predictions if they had a bit score of ≥50, *E*-value of ≤10^−3^, and average nucleotide identity of ≥70% across ≥2000 bp with the host genomes [25]. Finally, nucleotide sequences of vOTUs were compared to CRISPR spacers of MAGs using the sequence similarity method. The CRISPR spacers with >2 direct repeats in the array were identified using MinCED [103] and compared to nucleotide sequences of the vOTUs in this study. Hosts were selected if the spacers had zero mismatches to vOTUs.

The putative AMGs were identified and evaluated according to previously established methods [104]. Specifically, all brine vOTUs were processed with DRAM-v [105] to obtain gene functional annotations and identify AMGs. Genes on these contigs were regarded as AMGs if they had auxiliary scores ≤3 and the M flag. AMGs with transposon regions were not included. To obtain high-quality AMGs, and rule out AMGs from microbial contamination, CheckV (with default parameters) and manual inspection were then used to assess host-virus boundaries and remove the potential host fraction of the viral contig [106]. Next, a sequence of the viral AMG of interest (i.e., the e*psG* gene that is potentially involved in EPS synthesis) was subjected to further analyses to infer its evolutionary history. DIAMOND BLASTP [107] was used to query an AMG amino acid sequence against RefSeq database (release v99), in a sensitive mode with default settings, to obtain the reference sequences, i.e., the top 10 and 100 hits for the virus-encoded e*psG* gene sequence for conserved motif identification and phylogenetic analysis, respectively. In addition, microbe-encoded e*psG* genes were extracted from the CB microbial metagenomes [10] from the same project as this study and combined with previous sequences to study possible *epsG* gene transfers between viruses and their microbial hosts. Multiple sequence alignment was performed using MAFFT v.7.017 [108] with the E-INS-I strategy for 1000 iterations. The aligned sequences were then trimmed using TrimAl [109] with the flag gappyout. The substitution model was selected by ModelFinder [110] for accurate phylogenetic analysis. Phylogenies were generated using IQ-TREE [111] with 1000 bootstrap replicates, and then visualized in iTOL v5 [112]. Potential recombination among e*psG* genes was evaluated using nine programs: RDP [113], GENECONV [114], BootScan [115], MaxChi [116], Chimaera [117], SiScan [118], LARD [119], Phylpro [120], and 3Seq [121] within RDP5 [122]. A Bonferroni correction with a *p* value cut-off of 0.05 was applied in each of the tests. A sequence was considered a true recombinant if being supported by at least four of the nine programs. Branch and site selection pressure (*dN/dS*) analysis across lineages was carried out using codon models with maximum likelihood estimated with the codeml package in PAML [123] (Table S9). Visualization of the genome map for the virus containing the e*psG* gene was performed using Easyfig v2.2.5 [124]. Phage genes were identified by VirSorter [77].

Potential activity of brine viruses in CB and SB was investigated via recruiting sequencing reads (read identity of ≥95%; read coverage of ≥90%) of a CB and a SB metatranscriptome from the same project [10] to the 596 CB and 5084 SB vOTUs, using iVirus’ BowtieBatch and Read2RefMapper tools [83, 84]. A vOTU was considered as putative active if ≥50% of its genomic content was covered by the recruited metatranscriptomic reads, and thus was selected to assess viral quality via a rigorous inspection (as in identifying the AMG). Finally, 18 CB and 9 SB vOTUs were identified as potentially active in the brines.

Population genetics analyses, including SNP identification, microdiversity (*π* value) calculation, and the investigation of gene selection pressures were conducted with the tool MetaPop using the default parameters [53].

### Supplementary Information

**Additional file 1:** **Table S1.** Viromic statistics and viral concentrations of four samples. **Table S2.** Comparison in virus recovery from short- and long-read assemblies. **Table S3.** Size summary of vOTUs from CB17. The two assembly types (i.e., short+long-read assembly and short-read-only assesmbly) had identical sequencing depth, as described in **Table S2**. **Table S4.** Size summary of vOTUs from CB18. The two assembly types (i.e., short+long-read assembly and short-read-only assesmbly) had identical sequencing depth, as described in **Table S2**. **Table S5.** Taxonomic assignments and viral cluster summary. Viruses from this study, the NCBI RefSeq database, and 250 environmental metagenomes were included for clustering analysis. The environmental metagenomes included samples from below ecosystems: global oceans (GOV2 dataset), deep ocean water, deep ocean sediment, surfacer layer (top 1 meter) of permafrost (IsoGenie), soil, air, glacier cryoconite, glacier ice core, and lake water. **Table S6.** Viral cluster summary of the two vOTUs encoding fatty acid desaturase (FAD) genes. The two vOTUs are vOTU105_CB18_230153 and vOTU43_CB18_222366. Viral contigs that connected to above two vOTUs from Figure S4 and **Table S5** were used for network analysis in this Table. **Table S7.** Putative gene annotations of the 11,088 vOTUs from this study. Genes were annotated by comparing them to three databases PFAM, KEGG, and Uniref using DRAMv. **Table S8.** Gene annotation and phage gene identification of the vOTU vOTU4_CB17_43158 encoding the AMG espG.The methods used for gene annotation and phage gene identification are described in the legend of Fig. 5. **Table S9.** Tests for selection pressure of epsG gene using site and free-ratio models. **Table S10.** Summay and annotations of all brine viral genes under positive selection. The 23 putative phage tail fiber genes were highlighted in grey (i.e., Rows 3-25). **Table S11.** Scripts used for quality control of the short-read viromes based on DOE Joint Genome Institute's standards pipeline (Clum et al., 2021).

**Additional file 2: Figure S1.** Sampling site of Arctic cryopeg brine, sea-ice brine, and seawater near Utqiaġvik, Alaska. The CB samples were collected about 7 m below the permafrost surface (see Methods for more sampling details). SB and CB17 were sampled in 2017, while SW and CB18 were sampled in 2018. Abbreviations: CB, cryopeg brine; SB, sea-ice brine; SW, seawater. **Figure S2.** Rarefaction curves illustrate the changes of vOTU number across different sequencing depths in cryopeg brine, sea-ice brine, and seawater samples. **Figure S3.** Rank abundance curves of the top 100 abundant vOTUs in cryopeg brine samples from successive years (CB17 and CB18). The relative abundances of vOTUs (per each community) are ranked by their abundance in the sample CB17. **Figure S4.** Network clusters of viruses from this study (in green; A, CB; B, SB/SW), RefSeq database, and the 250 tested environmental metagenomes. Each node represents one viral genome/contig; the edge between nodes represents a significant relationship between two viral contigs/genomes with the shorter lengths accounting for stronger connection strength. The sources of viral contigs/genomes are indicated by colors. The details of VC clustering and statistical results are provided in Table S5. **Figure S5.** Community distributions of cryopeg brine, sea-ice brine, seawater, and GOV2 samples. Viruses in this study and the GOV2 dataset were combined and dereplicated to vOTUs, which were then used as baits to recruit the metagenomic reads generated in this study and GOV2 datasets to create an abundance table of all vOTUs (normalized to 1Gb of sequencing depth in each sample). Then the abundance table was used for generating a Bray Curtis distance matrix to visualize viral community distribution using a NMDS ordination. Sample types are indicated by colors. **Figure S6.** Phylogenetic tree of the *vEpsG *and *mEpsG* genes. The tree was inferred using maximum likelihood method with the EpsG protein sequences. Bootstrap values (expressed as percentages of 1000 replications) ≥40 are shown at the branch points. The scale bar indicates a distance of 1.0. The vEpsG sequence is indicated in red. The mEpsG sequences from CB microbial metagenomes [10] and NCBI nr database are indicated in purple and black, respectively. **Figure S7.** Multiple alignments of vEpsG and mEpsG protein sequences. The alignments include protein sequences from one *vEpsG* (numbered as 1), 11 brine *mEpsG* (numbered as 2–12), and the 10 closest *mEpsG* (to the *vEpsG*) from the NCBI nr database (numbered as 13–22). The protein sequences were aligned using MAFFT (v.7.458) with the E-INS-I strategy for 1000 iterations. The position numbers of aligned sequences are indicated at the top of alignments. The conserved motifs were identified by the tool MEME using default parameters and indicated by black boxes over the alignments. **Figure S8.** Comparisons of microdiversity among samples. (A) Genome-level microdiversity indicated by SNP density. (B) Percentage of genes that have at least one SNP. (C) Gene-level microdiversity indicated by SNP density.

## Data Availability

Long- and short-read viromic data have been deposited in the NCBI Sequence Read Archive under BioProject accession number PRJNA911607.

## Abbreviations

vOTU: Viral operational taxonomic unit
CB: Cryopeg brine
SB: Sea ice brine
SW: Seawater
VC: Viral cluster
HGT: Horizontal gene transfer
AMG: Auxiliary metabolic gene
HVR: Hypervariable region
SR: Short-read-only assembly
SLR: Short+long-read assembly
PCG: Percentage of complete genome
GOV2: Global ocean viromes 2.0
EPS: Extracellular polysaccharides
SNP: Single-nucleotide polymorphism
MAG: Metagenome-assembled genome
