# Supplementary material for: Lower viral evolutionary pressure under stable versus fluctuating conditions in subzero Arctic brines
Source: Microbiome. 2023 Aug 7;11:174. doi: 10.1186/s40168-023-01619-6 (PMC10405475; doi:10.1186/s40168-023-01619-6)
Supplement: Supplementary file 3 — Additional file 2: Figure S1. Sampling site of Arctic cryopeg brine, sea-ice brine, and seawater near Utqiaġvik, Alaska. The CB samples were collected about 7 m below the permafrost surface (see Methods for more sampling details). SB and CB17 were sampled in 2017, while SW and CB18 were sampled in 2018. Abbreviations: CB, cryopeg brine; SB, sea-ice brine; SW, seawater. Figure S2. Rarefaction curves illustrate the changes of vOTU number across different sequencing depths in cryopeg brine, sea-ice brine, and seawater samples. Figure S3. Rank abundance curves of the top 100 abundant vOTUs in cryopeg brine samples from successive years (CB17 and CB18). The relative abundances of vOTUs (per each community) are ranked by their abundance in the sample CB17. Figure S4. Network clusters of viruses from this study (in green; A, CB; B, SB/SW), RefSeq database, and the 250 tested environmental metagenomes. Each node represents one viral genome/contig; the edge between nodes represents a significant relationship between two viral contigs/genomes with the shorter lengths accounting for stronger connection strength. The sources of viral contigs/genomes are indicated by colors. The details of VC clustering and statistical results are provided in Table S5. Figure S5. Community distributions of cryopeg brine, sea-ice brine, seawater, and GOV2 samples. Viruses in this study and the GOV2 dataset were combined and dereplicated to vOTUs, which were then used as baits to recruit the metagenomic reads generated in this study and GOV2 datasets to create an abundance table of all vOTUs (normalized to 1Gb of sequencing depth in each sample). Then the abundance table was used for generating a Bray Curtis distance matrix to visualize viral community distribution using a NMDS ordination. Sample types are indicated by colors. Figure S6. Phylogenetic tree of the vEpsG and mEpsG genes. The tree was inferred using maximum likelihood method with the EpsG protein sequences. Bootstrap values (expressed [file 40168_2023_1619_MOESM2_ESM.docx]

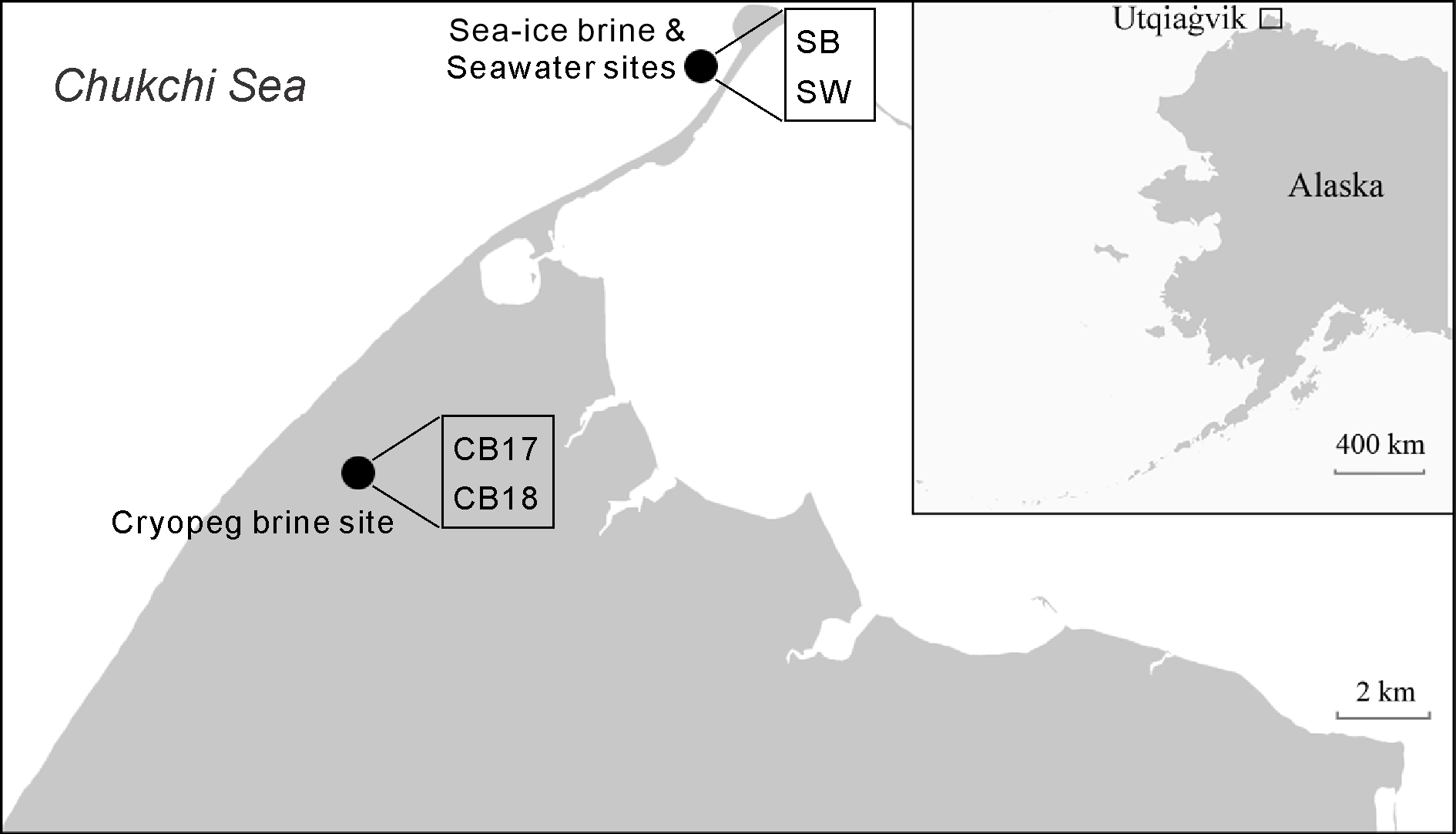


**Figure S1. Sampling site of Arctic cryopeg brine, sea-ice brine, and seawater near Utqiaġvik, Alaska.** The CB samples were collected about 7 m below the permafrost surface (see Methods for more sampling details). SB and CB17 were sampled in 2017, while SW and CB18 were sampled in 2018. Abbreviations: CB, cryopeg brine; SB, sea-ice brine; SW, seawater.


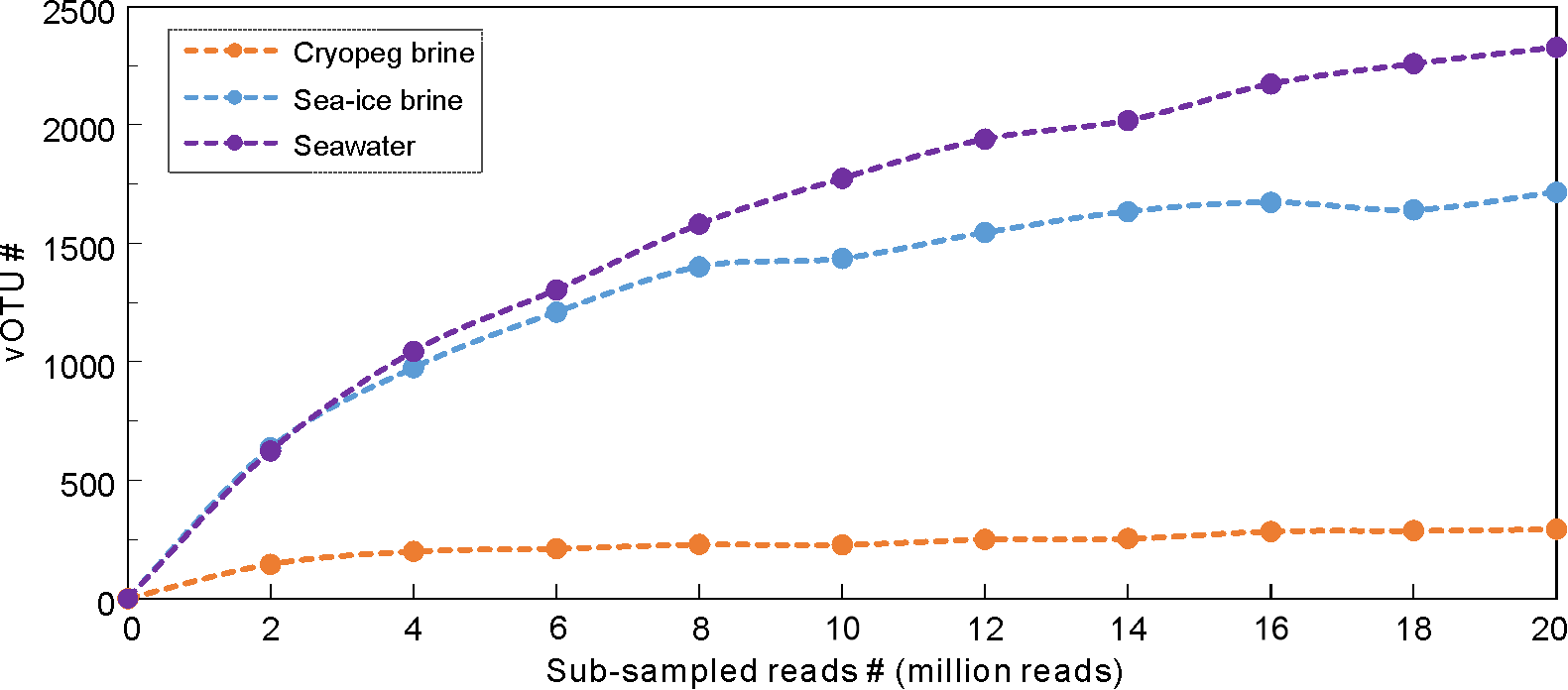


**Figure S2.** Rarefaction curves illustrate the changes of vOTU number across different sequencing depths in cryopeg brine, sea-ice brine, and seawater samples.


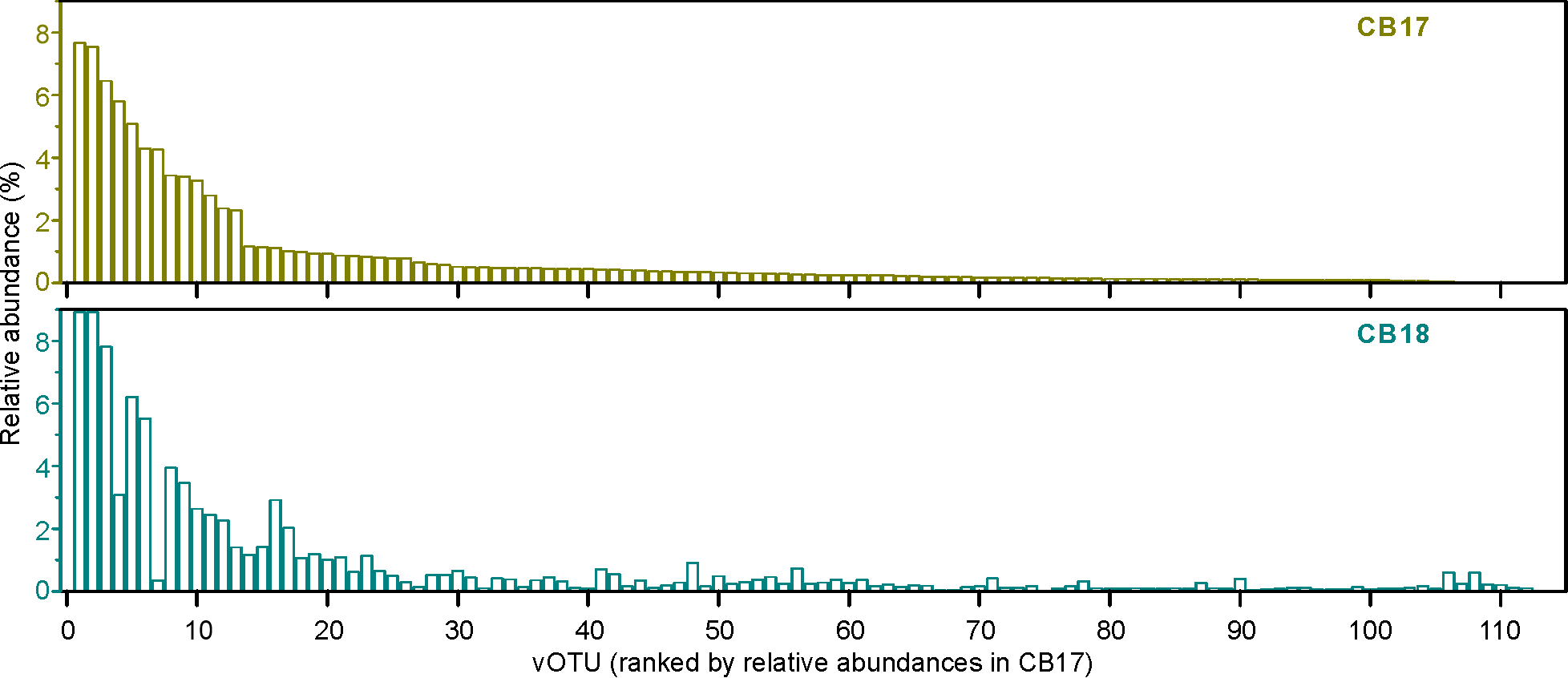


**Figure S3. Rank abundance curves of the top 100 abundant vOTUs in cryopeg brine samples from successive years (CB17 and CB18).** The relative abundances of vOTUs (per each community) are ranked by their abundance in the sample CB17.


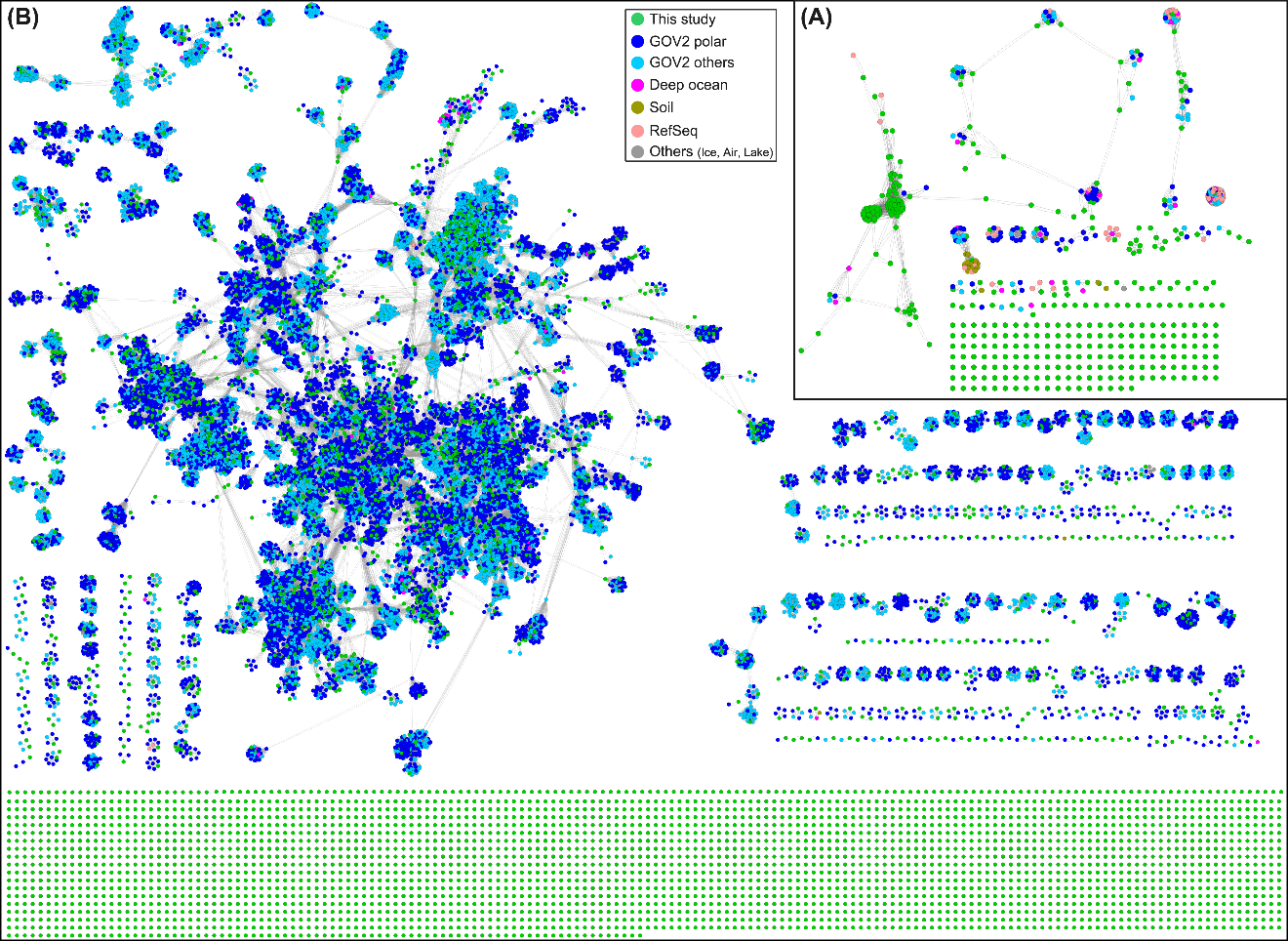


**Figure S4. Network clusters of viruses from this study (in green; A, CB; B, SB/SW), RefSeq database, and the 250 tested environmental metagenomes.** Each node represents one viral genome/contig; the edge between nodes represents a significant relationship between two viral contigs/genomes with the shorter lengths accounting for stronger connection strength. The sources of viral contigs/genomes are indicated by colors. The details of VC clustering and statistical results are provided in Table S5.


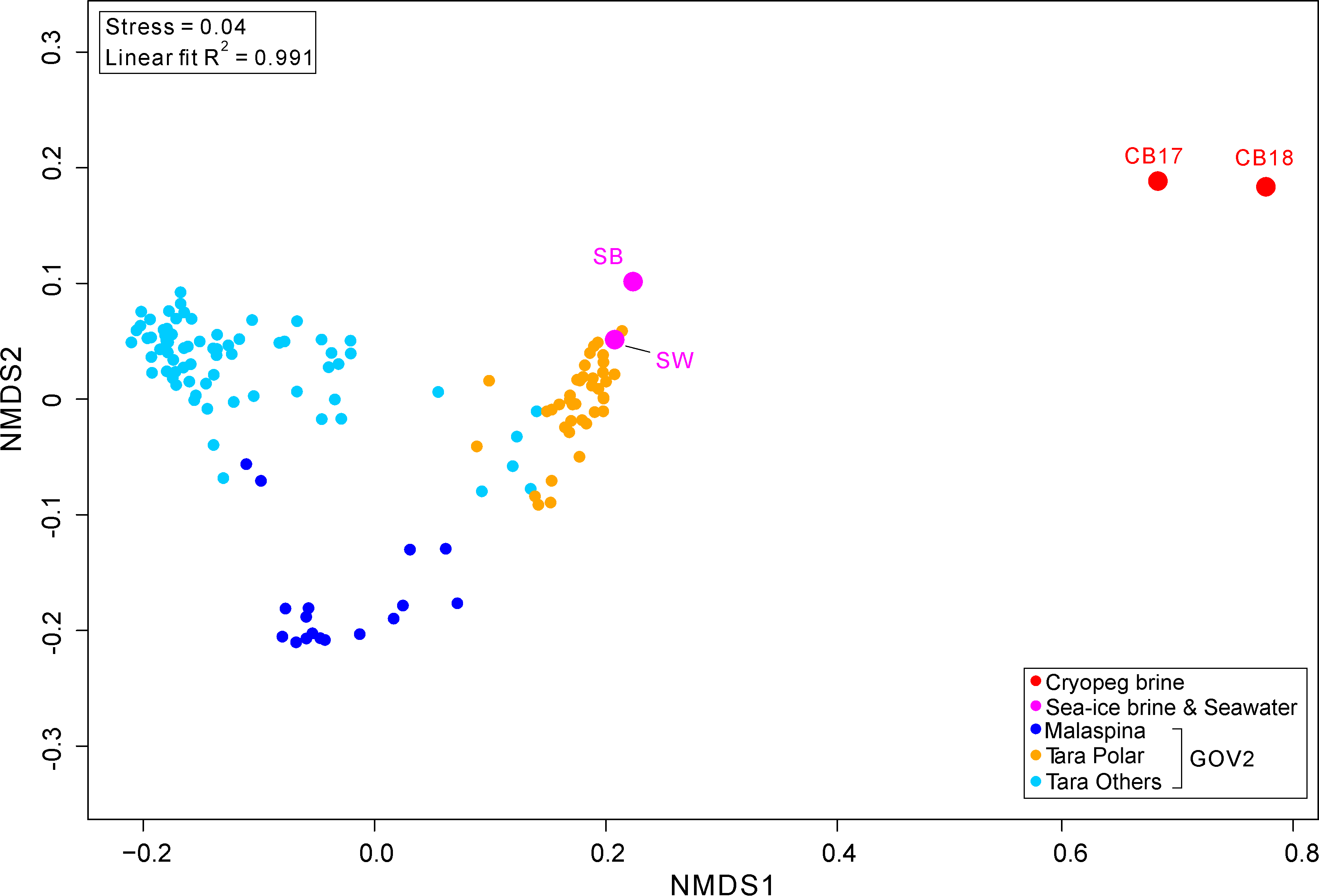


**Figure S5. Community distributions of cryopeg brine, sea-ice brine, seawater, and GOV2 samples.** Viruses in this study and the GOV2 dataset were combined and dereplicated to vOTUs, which were then used as baits to recruit the metagenomic reads generated in this study and GOV2 datasets to create an abundance table of all vOTUs (normalized to 1Gb of sequencing depth in each sample). Then the abundance table was used for generating a Bray Curtis distance matrix to visualize viral community distribution using a NMDS ordination. Sample types are indicated by colors.

**
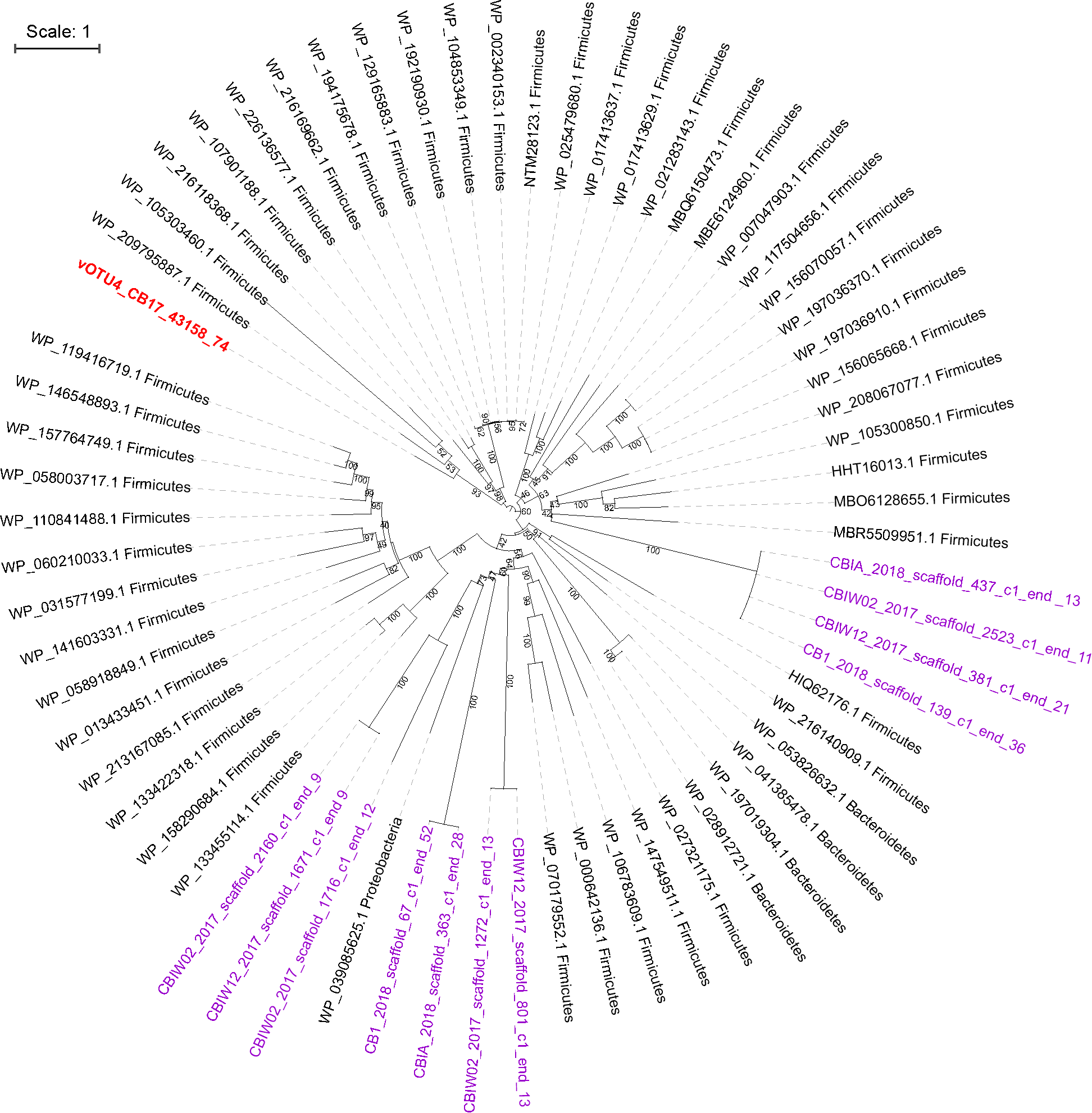
**

**Figure S6. Phylogenetic tree of the *vEpsG* and *mEpsG* genes.** The tree was inferred using maximum likelihood method with the EpsG protein sequences. Bootstrap values (expressed as percentages of 1000 replications) ≥40 are shown at the branch points. The scale bar indicates a distance of 1.0. The vEpsG sequence is indicated in red. The mEpsG sequences from CB microbial metagenomes [10] and NCBI nr database are indicated in purple and black, respectively.

**
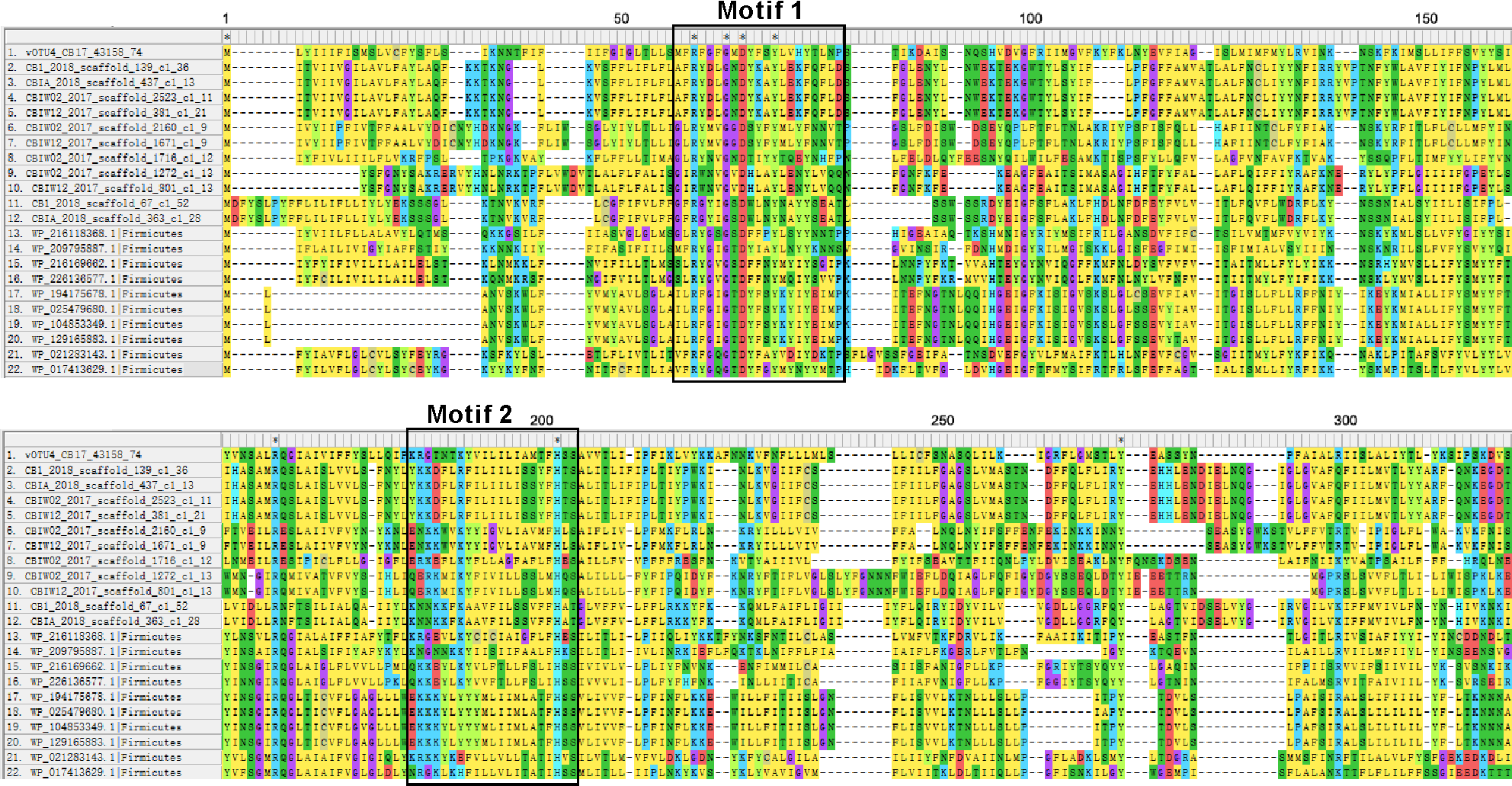
**

**
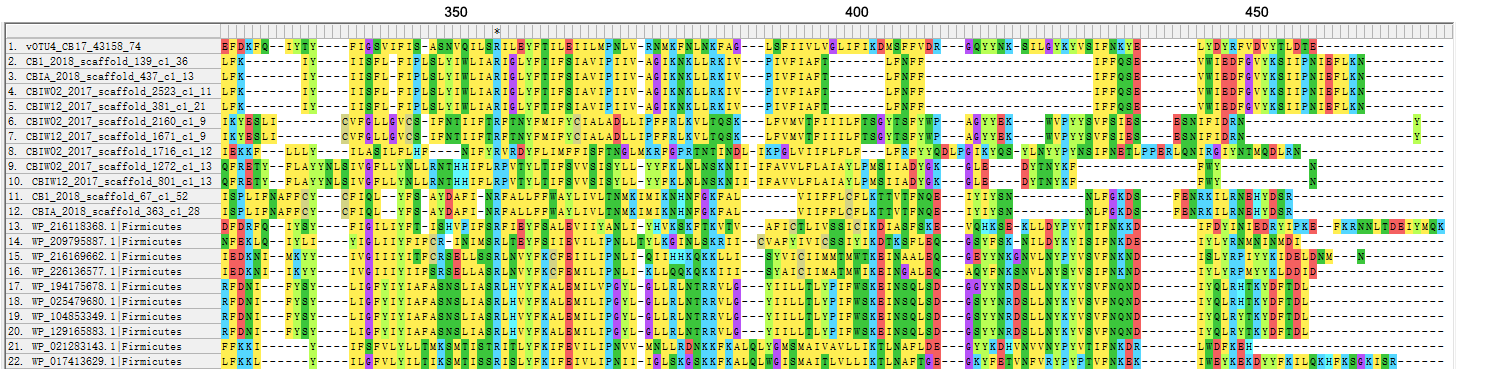
**

**Figure S7. Multiple alignments of vEpsG and mEpsG protein sequences.** The alignments include protein sequences from one *vEpsG* (numbered as 1), 11 brine *mEpsG* (numbered as 2–12), and the 10 closest *mEpsG* (to the *vEpsG*) from the NCBI nr database (numbered as 13–22). The protein sequences were aligned using MAFFT (v.7.458) with the E-INS-I strategy for 1000 iterations. The position numbers of aligned sequences are indicated at the top of alignments. The conserved motifs were identified by the tool MEME using default parameters and indicated by black boxes over the alignments.

**
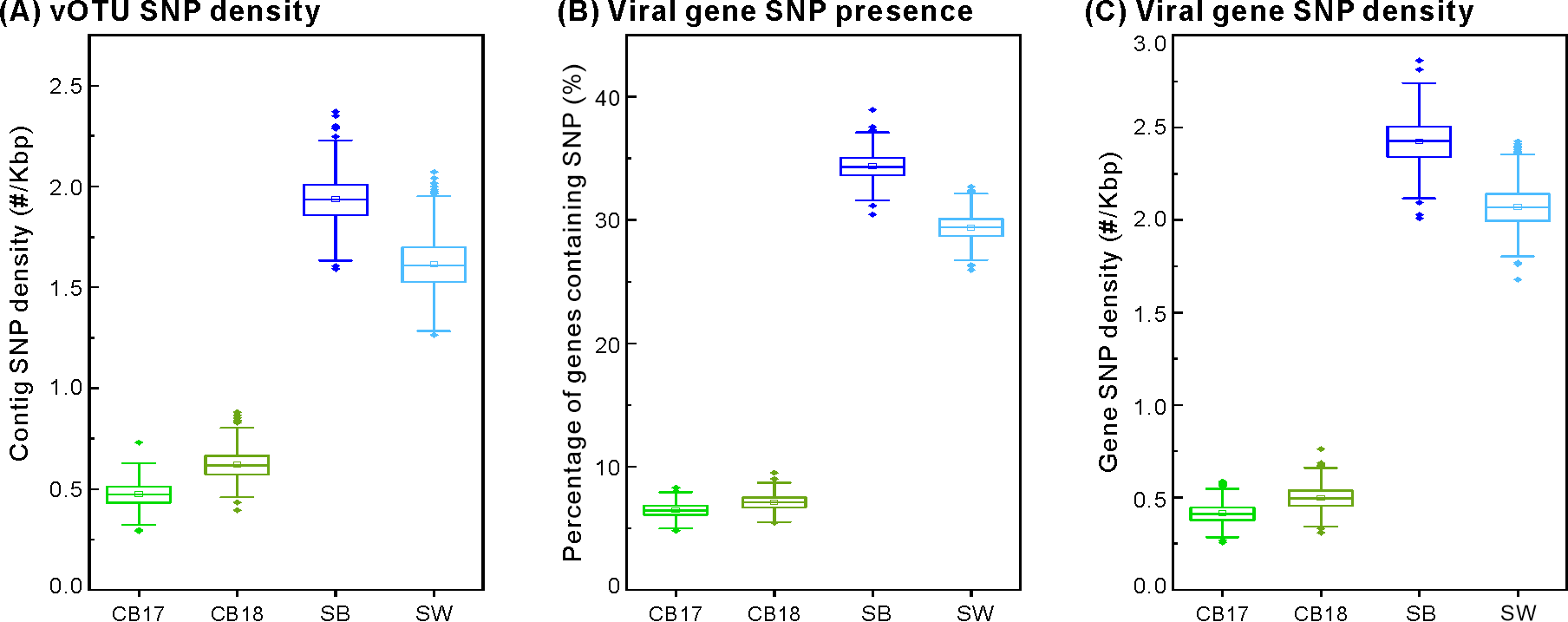
**

**Figure S8. Comparisons of microdiversity among samples.** (A) Genome-level microdiversity indicated by SNP density. (B) Percentage of genes that have at least one SNP. (C) Gene-level microdiversity indicated by SNP density.
